# Bit Error Rate Performance Improvement for Orthogonal Time Frequency Space Modulation with a Selective Decode-and-Forward Cooperative Communication Scenario in an Internet of Vehicles System

**DOI:** 10.3390/s24165324

**Published:** 2024-08-17

**Authors:** Selman Kulaç, Müjdat Şahin

**Affiliations:** Department of Electrical and Electronics Engineering, Faculty of Engineering, Duzce University, 81620 Duzce, Turkey; mujdat216998@ogr.duzce.edu.tr

**Keywords:** OTFS, SDF, BER analysis, cooperative communications, smart vehicles, IoT, IoV, V2X

## Abstract

Orthogonal time frequency space (OTFS) modulation has recently found its place in the literature as a much more effective waveform in time-varying channels. It is anticipated that OTFS will be widely used in the communications of smart vehicles, especially those considered within the scope of Internet of Things (IoT). There are efforts to obtain customized traditional point-to-point single-input single-output (SISO)-OTFS studies in the literature, but their BER performance seems a bit low. It is possible to use cooperative communications in order improve BER performance, but it is noticeable that there are very few OTFS studies in the area of cooperative communications. In this study, to the best of the authors’ knowledge, it is addressed for the first time in the literature that better performance is achieved for the OTFS waveform transmission in a selective decode-and-forward (SDF) cooperative communication scenario. In this context, by establishing a cooperative communication model consisting of a base station/source, a traffic sign/relay and a smart vehicle/destination moving at a constant speed, an end-to-end BER expression is derived. SNR-BER analysis is performed with this SDF-OTFS scheme and it is shown that a superior BER performance is achieved compared to the traditional point-to-point single-input single-output (SISO)-OTFS structure.

## 1. Introduction

Internet of Things (IoT) aims to connect objects, including drones, high-speed trains, mobile users and the like, at any time and place [1]. The growing spread of IoT, Internet of Vehicles (IoV), Vehicle-to-Vehicle (V2V) and Vehicle-to-Everything (V2X) communication technologies has received widespread attention. IoV, V2I, V2V and V2X technologies also have great potential as one of the main scenarios of next-generation mobile communication technologies. Multimedia transmission and transmission for autonomous driving can be considered as application examples within these technologies in intelligent vehicles, known as smart vehicles. In this context, significant improvements are needed in terms of ultra-high reliability and high quality of service. Formal methods used in the IoT environment for connected vehicle protocols can be found in [2,3,4].

IoT systems have to be able to operate in unpredictable mobile environments, such as moving vehicles [5]. Mobile communication technologies are developing according to growing demands. The demands for mobile IoT-integrated device communications are seen as important because it is predicted that the number of mobile IoT-integrated devices will increase, and a large portion of these devices are predicted to be smart vehicles, smart transport systems and smart traffic systems. Mobility-related issues are important in this scope [6].

Orthogonal frequency division multiplexing (OFDM) is a waveform commonly used in fourth-generation (4G) and fifth-generation (5G) cellular communication systems. OFDM has found its place in practice as a good solution against inter-symbol interference (ISI) effects that usually appear in wideband communication channels. But the Doppler effects caused by high mobility causes performance degradation in existing systems that include OFDM waveform transmissions in mobile communications. Time-varying channels because of mobile environments create inter-carrier interference (ICI) effects on OFDM waveforms. For this reason, a new appropriate waveform known as orthogonal time frequency space (OTFS) modulation has been developed, especially for time-varying channels. OTFS modulation has been developed to deal with time-varying fading as well as Doppler spread in channel under high mobility conditions. OTFS can transform the time-varying channel into one that is stable, separable in the delay-Doppler domain and that can bypass the fast-fading channel characteristic. At the same time, OTFS shifts the information in the delay-Doppler domain instead of the time–frequency domain to achieve complete time and frequency diversity. Additionally, channel sparsity in the delay-Doppler domain can also be utilized by OTFS to enhance system performance. OTFS is convenient for mobile communications especially in cases of high mobility conditions, as well as Internet of Things (IoT), ultra-reliable low-latency communications and advanced mobile broadband scenarios. Information symbols put in the delay-Doppler domain can be converted to the standard time–frequency domain used in conventional waveforms such as OFDM [2,7,8]. OTFS for vehicular networks (such as drones, high-speed trains and automobiles), OTFS for underwater acoustic communications, OTFS for non-terrestrial networks (such as LEO satellite communication networks), and OTFS for high-frequency-band (mmWave and THz bands) communications can be considered as practical examples or case studies of OTFS modulations [1].

One of the most important distortion effects on transmitted signals seen in the wireless channels is known as fading caused by multipath propagation. One of the techniques to overcome fading effects is to use multi-antenna systems like multi-input multi-output (MIMO) systems. This technique falls within the scope of antenna diversity. In recent years, research on MIMO structures and code design suitable for these structures has found wide coverage in the literature. Although transmitter antenna diversity methods are particularly convenient for base stations of cellular systems, they are not very suitable for mobile terminals in terms of size, cost and hardware complexity. In addition, the powerful shadowing effect caused by crowded vehicles and buildings can especially lead to serious performance loss in V2V and V2X communications. As a solution to this problem, the “cooperative diversity or cooperative communication” technique has been used. In cooperative communication, the source-transmitter provides transmitter diversity by sending its information directly to the destination-receiver through one or more cooperators (relays) [9]. Cooperative communication technology achieves cooperative diversity through relays, which can cope with the impact of shadowing effect and improve communication reliability. In this way, the effect of a less costly MIMO system with high efficiency is achieved [2].

One of the cooperative diversity methods in which users can act as relays is the “amplify and forward (AF)” method. It is a cooperative communication method first proposed by the authors of [10]. In accordance with this method, the other cooperating user amplifies and transmits the signal coming from the source user.

Another method, the “decode and forward (DF)” method, was first proposed by the authors of [11]. In accordance with this scenario, the other cooperative user decodes the signal coming from the source user and transmits it digitally again.

Another method, “coded cooperation”, was first proposed by the authors of [12]. In accordance with this proposed method, the signal coming from the source user is re-encoded by another auxiliary user and sent to the destination.

In the “selection relaying” method mentioned in [13], the focus is on the amplifier gain, which depends on the fading coefficient between the source and the relay. If the measured gain is below a specific threshold, the source goes on its transmission to the destination. If the measured gain is above the threshold, the relay transmits using AF or DF to achieve diversity gain.

In [14], the relay remains silent when the S-R link is in outage and assists only when the mutual information involving the relay self-interference is greater than R bit/s/Hz, i.e., when the outage does not occur.

Previous works such as [15,16] have emphasized the performance degradation of terminal mobility on cooperative communication. On the other hand, this problem can be reduced by exploiting the robustness of OTFS in cooperative communication [2,17,18,19,20,21]. For example, in the study [2], the proposed cooperative OTFS system with AF and DF protocols achieved gains of approximately 15.1 dB and 12.0 dB, respectively, compared to the non-cooperative OTFS system. The proposed cooperative OTFS system beats the traditional non-cooperative OTFS system as better diversity gains and end-to-end SNR can be obtained with the help of relaying. In [17], the authors express that the natural robustness of OTFS can mitigate the presence of the node mobility problem in collaborative communications. Therefore, they present an analysis of the end-to-end performance of MIMO-OTFS in decode-and-forward (DaF) cooperative systems. In [18], the authors highlight making cooperative communication an attractive model to overcome the restrictions of conventional point-to-point OTFS systems. In this context, they present an end-to-end performance analysis of OTFS index modulation in the DaF cooperative system. In [20], it is stated that OFDM systems put limitations such as sensitivity to the Doppler effect in high-speed mobile conditions. To solve this problem, OTFS-assisted cooperative transmission (CT) for UAV swarms is proposed, which can overcome the above-mentioned limitations. In the study [21], the presence of cooperative UAV transmitting jamming signals using OTFS modulation was shown to enhance the security of the legitimate LEO SatCom link. The study [22] emphasizes that UAV cooperation can perform significantly better than a non-cooperative case with respect to the probability of outage of the OTFS-based LEO-SatCom transmission. Therefore, there is a growing attention in understanding the performance of OTFS in relay systems under high mobility circumstances.

Selective DF protocol-based cooperative communication, wherein the relay retransmits the symbol to the destination on the condition that the current SNR at the relay is greater than a threshold, is considered in [12]. The paper expresses the end-to-end performance of a selective DF-based MIMO-OSTBC (Orthogonal Space Time Block Code) collaborative wireless system over time-varying fading channels. In [23], better end-to-end error performance was obtained using MIMO and STBC together with SDF protocol when compared to AF and DF protocols. In [24], it is expressed that the SDF-based collaboration protocol can be used with MIMO techniques; to further improve the PEP performance of the cooperative system, STBC and maxima rate combining (MRC) are used. In study [25], it was stated that, compared to the DF protocol, SDF-supported relays can prevent the transmission of non-correct information to destinations to increase system performance. It has been emphasized that the SDF protocol is advantageous over its classical DF counterparts. As mentioned above, SDF protocol is a recently used method in cooperative communication [15,23,24,25]. In our work, we have also improved the system performance by using OTFS modulation in the SDF protocol or scheme where the receiver part is mobile.

There are efforts to obtain customized traditional point-to-point single-input single-output (SISO)-OTFS studies in the literature, but their BER performance seems rather low. It is possible to use cooperative communications in order improve BER performance, but it is noticeable that there are very few OTFS studies in the area of cooperative communications. To the best of our knowledge, we also address for the first time in the literature that the bit error rate (BER) performance of a mobile receiver is improved by using the selective decoding-and-forwarding (SDF) scheme in combination with OTFS transmissions in the scope of cooperative communications.

## 2. System Model

**Figure 1:**
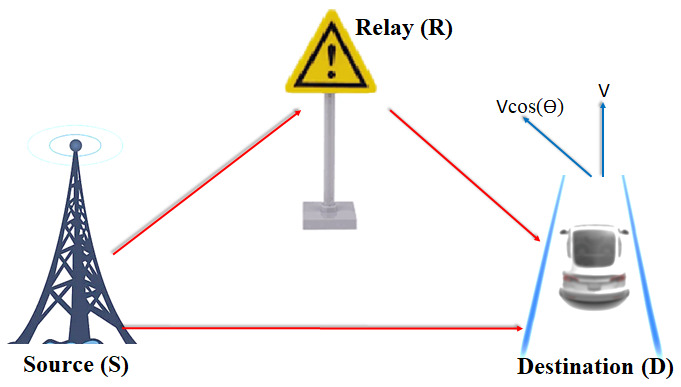
System model of the SDF-OTFS system.

In this study, a single relay structure is taken into account. In our system model, there is a base station as a source, a traffic sign as a relay and a moving vehicle as a destination as seen in Figure 1. The direction of the moving vehicle is perpendicular to the source. This system model is very practical, because today it is frequently encountered base stations and traffic signs on the roads within the scope of 4G/5G cellular communication systems. In this scenario, only a base station and a traffic sign with a receiver/transmitter feature are sufficient. Therefore, this model is simply considered.

The focus is on the performance improvement of the data (internet) connection when a moving vehicle communicates with the base station. When the bit error rate (BER) in the transmissions increase, interruptions or outages in the data (internet) connection also increase. This system model is proposed in order to minimize BER.

In this system model, transmissions are performed in two time slots in a downlink scenario. These time slots are expressed as transmission phases. Different protocols can be used when the relay processes the signal. In this proposed method, an SDF protocol or scheme is used, and this SDF protocol runs in two phases. In the first phase, the source broadcasts the symbol and the symbol is received by the destination and the relay. In the second phase, only if the code (symbol) transmitted by the source is correctly decoded in the relay does the relay then retransmit to the receiver/destination.

### 2.1. Phase 1: Source Broadcasts Symbol to Destination and Relay (for Destination)

The source broadcasts to the receiver (destination) and relay in this phase. It is assumed that there are two paths in each transmission in this system model. One of them is the line-of-sight (LOS) path and the other is the reflected path. In this phase, the direct path from the source to the destination (*s*,*d*) is considered as the LOS path, and the path from the source to the relay and then to the destination is considered as the reflected path (*s*,*r*,*d*).

For the destination, the received time domain signal is expressed as the point-to-point single-input single-output (SISO) model, as follows:

(1) $$r_{d}\left(t\right)=h_{s,d}s\left(t-\tau_{s,d}\right)+\;h_{s,r,d}e^{j2\pi \upsilon_{s,r,d}\left(t-\tau_{s,r,d}\right)}s\left(t-\tau_{s,r,d}\right)$$

where $h_{s,d}$ is the channel gain of the LoS path, $\tau_{s.d}$ is the delay shift of the LoS path, s(t) is the transmitted signal from the source, $h_{s,r,d}$ is the channel gain of the NLoS (reflected) path, $\nu_{s,r,d}=f_{c}\frac{vCos\theta }{c}=\upsilon_{r,d}$ is the Doppler frequency (shift) of the reflected path and $\tau_{s,r,d}$ is the delay shift of the reflected path.

For two propagation paths, a specific 2-tap doubly selective time-variant channel impulse response (CIR) for this transmission is given below. When s(t) passes through this channel, Equation (1) is obtained:

(2) $$g\left(t,\tau \right)=h_{s,d}\delta \left(t-\tau_{s,d}\right)+\;h_{s,r,d}e^{j2\pi \upsilon_{s,r,d}\left(t-\tau_{s,r,d}\right)}\delta \left(t-\tau_{s,r,d}\right)$$

To make the mathematical expressions for the time-varying channel above easier to understand, we present below the received linearly modeled signal from the source to the destination, in time domain, in vector and matrix forms in the context of OTFS modulation:

(3) $${\bar{r}}_{s,d}=H_{s,d}\bar{s}+\bar{n}$$

where $\bar{s}$ is the transmitted signal vector in time domain and $\bar{n}$ is the additive Gaussian noise vector.

The general mathematical expression of the channel matrix $H_{s,d}$ is expressed as

(4) $$H_{s,d}=\sum_{i=1}^{P}h_{i}\Pi_{MN}^{l_{i}}\Delta_{MN}^{k_{i}}$$

where P is number of channel paths, *l_i_* = (*M*Δ*f*)*τ_i_* represents the delay taps (bins as integer numbers), *k_i_* = (*N*/Δ*f*)*γ_i_* represents the Doppler taps (bins as integer numbers), Δ*f* is subcarrier spacing, M is the number of subcarriers and N is the size of the time slots. $\Pi_{MN}^{l_{i}}$ denotes the MN × MN cyclic-shift matrix while $\Delta_{MN}^{k_{i}}$ denotes the MNxMN diagonal matrix, and they are given below, respectively.

(5) $$\Pi_{MN}^{l_{i}}\;\;≜\;\left[\begin{array}{cccc} 0 & \ldots & 0 & 1\\ 1 & \cdots & 0 & 0\\ \vdots & \ddots & \vdots & \vdots \\ 0 & \cdots & 1 & 0 \end{array}\right]$$

(6) $$\Delta_{MN}^{k_{i}}≜diag\left[z^{0}\;,\;z^{1}\;,\;\ldots ,\;z^{\left(n-1\right)}\right]\;\mathrm{with}\;\;\;\;z\;≜\;e^{\left(\frac{j2\pi }{MN}\right)}.$$

In vectorized form, the received signal from the source to destination in the delay-Doppler domain can be written as

(7) $${\bar{y}}_{s,d}=H_{{eff}_{s,d}}\bar{x}+\bar{\tilde{n}}$$

where $H_{{eff}_{s,d}}=\left(F_{N}⊗G_{rx}\right)H_{s,d}\left(F_{N}^{H}⊗G_{tx}\right)$ indicates an effective channel matrix, $\bar{x}$ is the transmitted signal in the delay-Doppler domain, and $\bar{\tilde{n}}=\left(F_{N}⊗G_{rx}\right)\bar{n}$ is the noise vector. $F_{N}$ and $F_{N}^{H}$ denote the N-point FFT matrix and the N-point IFFT matrix, respectively.

For the rectangular waveforms, $H_{{eff}_{s.d}}^{rect}=\left(F_{N}⊗I_{M}\right)H_{s,d}\left(F_{N}^{H}⊗I_{M}\right)$ is obtained using $G_{rx}=G_{tx}=I_{M}$. Then, Equation (7) can be transformed below.

(8) $${\bar{y}}_{s,d}=H_{{eff}_{s.d}}^{rect}\bar{x}+\bar{\tilde{n}}$$

First Path (LoS Path): Since the vehicle moves in the *V* direction, the receiver moves perpendicular to the source. The first Path (LoS path) is the shortest path for i = 1. There is no delay or Doppler shift in that channel. Therefore, the channel with zero delay and Doppler in the first channel case, as presented in [24], and *l*_1_ = 0, *k*_1_ = 0 are obtained.

Since the movement direction of our vehicle is in the *V* direction, it moves perpendicularly to the source, as we stated before. For this reason, the velocity vector value along the LoS path line is zero. Since there is no velocity component in the source direction, the Doppler effect for the LoS path is zero, and therefore, the $\upsilon_{i}$ value will be zero (very low). Therefore, the *k_i_* value is taken as 0.

Since the LoS path is the shortest path, the delay value will be minimum and the $\tau_{i}$ value will be low. Therefore, the *l_i_* value can be accepted as 0.

Second Path (NLoS Path–Reflected Path): Since the vehicle moves in the *V* direction, the receiver moves perpendicular to the source. However, there is a velocity component in the relay direction. Additionally, path 2 (NLoS path) is the longest path for i = 2. There are delay and Doppler shifts in that channel. Therefore, the channel with both delay and Doppler in the fourth channel case, presented in [22], and *l*_2_* = *1, *k*_2_ = 1 are obtained.

Since the movement direction of our vehicle is in the *V* direction, it moves perpendicularly to the source, as we stated before. For this reason, the velocity vector component occurs along the NLoS path. Since there is a velocity component in the relay direction, the Doppler effect also occurs for the NLoS path, and therefore, the $\upsilon_{i}$ value will be greater than zero. Therefore, the *k_i_* value can be accepted as 1.

Since the NLoS path is the longest, both the delay value and the τi value will be high. Therefore, the value of *l_i_* can be accepted as 1.

According to our model, as expressed above, P = 2, and $k_{i}=\left[\begin{array}{cc} 0 & 1 \end{array}\right]\;and\;l_{i}=\left[\begin{array}{cc} 0 & 1 \end{array}\right]$ is defined for Phase 1 for the destination.

When M = 2 and N = 2 are taken into account, then Equation (4) is transformed to

(9) $$H_{s,d}=\sum_{i=1}^{2}h_{i}\Pi_{MN}^{li}\Delta_{MN}^{ki}$$

When i = 1, then $H_{1_{s,d}}=h_{1}\Pi^{0}\Delta^{0}$ is obtained, where

(10) $$\Pi^{0}=\left[\begin{array}{cccc} 1 & 0 & 0 & 0\\ 0 & 1 & 0 & 0\\ 0 & 0 & 1 & 0\\ 0 & 0 & 0 & 1 \end{array}\right],\;\Delta^{0}=\left[\begin{array}{cccc} 1 & 0 & 0 & 0\\ 0 & 1 & 0 & 0\\ 0 & 0 & 1 & 0\\ 0 & 0 & 0 & 1 \end{array}\right]$$

Substituting $\Pi^{0}$ and $\Delta^{0}$ in the equation, the $H_{1_{s,d}}$ channel matrix is obtained,

(11) $$H_{1_{s,d}}=\;\left[\begin{array}{cccc} h_{1} & 0 & 0 & 0\\ 0 & h_{1} & 0 & 0\\ 0 & 0 & h_{1} & 0\\ 0 & 0 & 0 & h_{1} \end{array}\right]$$

When i = 2, $H_{2_{s,d}}=h_{2}\Pi^{1}\Delta^{1}$ is obtained, where

(12) $$\Pi^{1}=\left[\begin{array}{cccc} 0 & 0 & 0 & 1\\ 1 & 0 & 0 & 0\\ 0 & 1 & 0 & 0\;\\ 0 & 0 & 1 & 0 \end{array}\right],\;\Delta^{1}=\left[\begin{array}{cccc} 1 & 0 & 0 & 0\\ 0 & z & 0 & 0\\ 0 & 0 & z^{2} & 0\\ 0 & 0 & 0 & z^{3} \end{array}\right]$$

(13) $$z={\mathrm{e}}^{j\frac{2\Pi }{MN}}={\mathrm{e}}^{j\frac{\Pi }{2}}=j$$

(14) $$\Delta^{1}=\left[\begin{array}{cccc} 1 & 0 & 0 & 0\\ 0 & j & 0 & 0\\ 0 & 0 & -1 & 0\\ 0 & 0 & 0 & -j \end{array}\right]$$

(15) $$H_{2_{s,d}}=\left[\begin{array}{cccc} 0 & 0 & 0 & -h_{2}j\\ h_{2} & 0 & 0 & 0\\ 0 & h_{2}j & 0 & 0\\ 0 & 0 & {-h}_{2} & 0 \end{array}\right]$$

Since $H_{s,d}=H_{1_{s,d}}+H_{2_{s,d}}$, we sum the matrices $H_{1_{s,d}}$ and $H_{2_{s,d}}$, and then we obtain the $H_{s,d}$ matrice:

(16) $$H_{s,d}=\left[\begin{array}{cccc} h_{1} & 0 & 0 & -h_{2}j\\ h_{2} & h_{1} & 0 & 0\\ 0 & h_{2}j & h_{1} & 0\\ 0 & 0 & {-h}_{2} & h_{1} \end{array}\right]$$

(17) $$H_{{eff}_{s,d}}^{rect}=\left(F_{N}⊗I_{M}\right)H_{s,d}\left(F_{N}^{H}⊗I_{M}\right)$$

(18) $${\left[F_{N}\right]}_{k,l\to (N-1)}\;=\{\frac{1}{\sqrt{N}}{\mathrm{e}}^{j\frac{2\Pi kl}{N}}\}$$

(19) $$F_{N}=\;\frac{1}{\sqrt{2}}\left[\begin{array}{cc} 1 & 1\\ 1 & e^{j\Pi } \end{array}\right]=\frac{1}{\sqrt{2}}\left[\begin{array}{cc} 1 & 1\\ 1 & -1 \end{array}\right]$$

(20) $$F_{N}^{H}=\frac{1}{\sqrt{2}}\left[\begin{array}{cc} 1 & 1\\ 1 & -1 \end{array}\right],\;I_{M}=\;\left[\begin{array}{cc} 1 & 0\\ 0 & 1 \end{array}\right]$$

(21) $$F_{N}^{H\;}⊗\;I_{M}=F_{N}⊗\;I_{M}\;=\frac{1}{\sqrt{2}}\left[\begin{array}{cc} 1 & 1\\ 1 & -1 \end{array}\right]⊗\;\left[\begin{array}{cc} 1 & 0\\ 0 & 1 \end{array}\right]\;=\frac{1}{\sqrt{2}}\left[\begin{array}{cccc} 1 & 0 & 1 & 0\\ 0 & 1 & 0 & 1\\ 1 & 0 & -1 & 0\\ 0 & 1 & 0 & -1 \end{array}\right]$$

(22) $$H_{{eff}_{s.d}}^{rect}=\frac{1}{\sqrt{2}}\left[\begin{array}{cccc} 1 & 0 & 1 & 0\\ 0 & 1 & 0 & 1\\ 1 & 0 & -1 & 0\\ 0 & 1 & 0 & -1 \end{array}\right]\;\times \left[\begin{array}{cccc} h_{1} & 0 & 0 & -h_{2}j\\ h_{2} & h_{1} & 0 & 0\\ 0 & h_{2}j & h_{1} & 0\\ 0 & 0 & -h_{2} & h_{1} \end{array}\right]\;\times \frac{1}{\sqrt{2}}\left[\begin{array}{cccc} 1 & 0 & 1 & 0\\ 0 & 1 & 0 & 1\\ 1 & 0 & {\;z}^{-2} & 0\\ 0 & 1 & 0 & z^{-2} \end{array}\right]$$

(23) $$H_{{eff}_{s.d}}^{rect}=\left[\begin{array}{cccc} h_{1} & 0 & 0 & h_{2}j\\ 0 & h_{1} & h_{2} & 0\\ 0 & -h_{2}j & h_{1} & 0\\ h_{2} & 0 & 0 & h_{1} \end{array}\right]$$

### 2.2. Phase 1: Source Broadcasts Symbol to Destination and Relay (for Relay)

The source broadcasts to the receiver (destination) and relay. For the relay, the received time domain signal is expressed as the point-to-point SISO model as follows:

(24) $$r_{r}\left(t\right)=h_{s,r}s\left(t-\tau_{+s,r}\right)+\;h_{s,d,r}e^{j2\pi \upsilon_{s,,d,r}\left(t-\tau_{s,d,r}\right)}s\left(t-\tau_{s,d,r}\right)$$

where $h_{s,r}$ is the channel gain of the LoS path, the Doppler frequency (shift) of the LoS path = 0, $\tau_{s,r}$ is the delay shift of the LoS path, s(t) is the transmitted signal from the source, $h_{s,d,r}$ is the channel gain of the NLoS (reflected) path, $\nu_{s,d,r}$ is the Doppler frequency of the NLoS path, and $\tau_{s,d,r}$ is the delay of the NLoS path.

For two propagation paths, a specific 2-tap doubly selective time-variant channel impulse response (CIR) for this transmission is given below. When s(t) passes through this channel, Equation (24) is obtained.

(25) $$g\left(t,\tau \right)=h_{s,r}\delta \left(t-\tau_{s,r}\right)+\;h_{s,d,r}e^{j2\pi \upsilon_{s,d,r}\left(t-\tau_{s,d,r}\right)}\delta \left(t-\tau_{s,d,r}\right)$$

To make the mathematical expressions for the time-varying channel above easier to understand, we present below the received signal from the source to the relay in the time domain in vector and matrix forms, in the context of OTFS modulation:

(26) $${\bar{r}}_{s,r}=H_{s,r}\bar{s}+\bar{n}$$

The general mathematical expression of the channel matrix $H_{s,r}$, effective channel matrix $H_{{eff}_{s,r}}^{rect}$ and vectorized form of the received signal in the delay-Doppler domain are similarly obtained using Equations (4)–(8).

First Path (LoS Path): Path 1 (LoS path) is the shortest path for i = 1. Since the relay is stationary, the Doppler effect for the LoS path is zero, and therefore, the value of $\upsilon_{i}$ will be zero (very low). Therefore, *k*_1_ = 0 is obtained.

Since the LoS path is the shortest path, the delay value will be minimum and $\tau_{i}$ will be low. Therefore, *l*_1_* = *0 is obtained.

Second Path (NLoS Path): Since the vehicle is moving in the V direction, there is a velocity component in the relay direction. Additionally, path 2 (NLoS path) is the longest path for i = 2. Therefore, the channel with both delay and Doppler in the fourth channel case, presented in [22], and *l*_2_* = *1, *k*_2_ = 1 are obtained. Since the direction of motion of our vehicle is in the V direction, there is a velocity component in the relay direction as mentioned before. For this reason, the velocity vector component occurs along the NLoS path. Since there is a velocity component in the relay direction, the Doppler effect also occurs for the NLoS path, and therefore, the value of $\upsilon_{i}$ will be greater than zero. Therefore, the value of $k_{i}$ can be considered as 1.

Since the NLoS path is the longest path, both the delay value and the τi value will be high. Therefore, the value of li can be considered as 1.

For LoS and NLoS paths, the values of $k_{i}$ and $l_{i}$ are 0 for i = 1 and 1 for i = 2 since they are the same for the source–destination scenario. Therefore, the channel matrix $H_{s,r}$ and the effective channel matrix $H_{{eff}_{s,r}}^{rect}$ can be obtained from the equations written above for source–destination Equations (9)–(23).

In vectorized form, the received signal from the source to the relay in the delay-Doppler domain can be written for rectangular waveforms:

(27) $${\bar{y}}_{s,r}=H_{{eff}_{s.r}}^{rect}\bar{x}+\bar{\tilde{n}}$$

### 2.3. Phase 2: Relay Retransmits the Symbol to Destination

The relay afterwards retransmits to the destination only if it is able to correctly decode within the SDF scheme. For the destination, the received time domain signal is expressed as a point-to-point SISO as follows:

(28) $$r_{d}\left(t\right)=h_{r,d}e^{j2\pi \upsilon_{r,d}\left(t-\tau_{r,d}\right)}s\left(t-\tau_{r,d}\right)+\;h_{r,s,d}s(t-\tau_{r,s,d})$$

where $h_{r,d}$ is the channel gain of the LoS path, $\upsilon_{r,d}$ is the Doppler frequency (shift) of the LoS path, s(t) is the transmitted signal from the source, $\tau_{r,d}$ is the delay shift of the LoS path, $h_{r,s,d}$ is the channel gain of the NLoS (reflected) path, and $\tau_{r,s,d}$ is the delay shift of the NLoS path.

The time-variant channel impulse response (CIR) for this transmission is given below:

(29) $$g_{\left(t,\upsilon \right)}=h_{r,d}e^{j2\pi \upsilon_{r,d}\left(t-\upsilon_{r,d}\right)}\delta \left(t-\tau_{r,d}\right)+\;h_{r,s,d}\delta \left(t-\tau_{r,s,d}\right)$$

To make the mathematical expressions for the time-varying channel above easier to understand, we present below the received signal from the relay to the destination in the time domain in vector and matrix forms, in the context of OTFS modulation:

(30) $${\bar{r}}_{r,d}=H_{r,d}\bar{s}+\bar{n}$$

First Path (LoS Path): Since the vehicle is moving in V direction, the receiver has a velocity component in the direction of the relay. Path 1 (LoS path) is the shortest path for i = 1. Therefore, the channel with zero delay and one Doppler in the third channel case, as presented in [22], and *l*_1_* = *0, *k*_1_ = 1 are obtained.

Since the direction of motion of our vehicle is V, there is a velocity component in the direction of the relay as mentioned before. Since there is a velocity component in the relay direction, the Doppler effect for the LoS path will be greater than zero, and therefore, the value of $\upsilon_{i}$ will be greater than zero. Therefore, the value of *k_i_* is taken as 1.

Since the LoS path is the shortest path, the delay value will be minimal and $\tau_{i}$ will be low. Therefore, the value of *l_i_* can be taken as 0.

Second Path (NLoS Path): In this scenario, the source acts as a reflector as it reflects the signal sent by the relay, which retransmits the incoming signal with the SDF protocol.

Since the vehicle moves in the V direction, the receiver moves perpendicularly to the source (relay). Path 2 (NLoS path) is the longest path for i = 2. Therefore, the channel with one delay and zero Doppler in the second channel case, as presented in [24], and *l*_2_* = *1, *k*_2_ = 0 are obtained. Since the direction of movement of our vehicle is in the V direction, it moves perpendicularly to the relay as we mentioned before. Therefore, the velocity vector component along the NLoS path is 0. Since there is no velocity component in the source direction, the Doppler effect for the NLoS path is zero, and therefore, the value of $\upsilon_{i}$ will be zero (very low). Therefore, the value of *k_i_* is taken as 0.

Since the NLoS path is the longest path, the delay value will be maximum and the $\tau_{i}$ value will be high. Therefore, the value of *l_i_* can be taken as 1.

According to our model, as expressed above, P = 2, and $k_{i}=\left[\begin{array}{cc} 1 & 0 \end{array}\right]\;and\;l_{i}=\left[\begin{array}{cc} 0 & 1 \end{array}\right]$ is defined for Phase 2 for the destination.

When M = 2 and N = 2 are taken into account, then Equation (4) is transformed into

(31) $$H_{r,d}=\sum_{i=1}^{2}h_{i}\Pi_{MN}^{li}\Delta_{MN}^{ki}$$

(32) $${{H_{r,d}}_{\;=}h}_{1}\Pi^{0}\Delta^{1}+h_{2}\Pi^{1}\Delta^{0}$$

When i = 1, then $H_{1_{r,d}}=h_{1}\Pi^{0}\Delta^{1}$ is obtained and i = 2; moreover, $H_{2_{r,d}}=h_{2}\Pi^{1}\Delta^{0}$ is obtained, where

(33) $$h_{1}\begin{array}{c} \Pi^{0}\\ \left[\begin{array}{cccc} 1 & 0 & 0 & 0\\ 0 & 1 & 0 & 0\\ 0 & 0 & 1 & 0\\ 0 & 0 & 0 & 1 \end{array}\right] \end{array}\times \begin{array}{c} \Delta^{1}\\ \left[\begin{array}{cccc} 1 & 0 & 0 & 0\\ 0 & j & 0 & 0\\ 0 & 0 & -1 & 0\\ 0 & 0 & 0 & -j \end{array}\right] \end{array}+\;h_{2}\begin{array}{c} \Pi^{1}\\ \left[\begin{array}{cccc} 0 & 0 & 0 & 1\\ 1 & 0 & 0 & 0\\ 0 & 1 & 0 & 0\;\\ 0 & 0 & 1 & 0 \end{array}\right]\times \end{array}\begin{array}{c} \Delta^{0}\\ \left[\begin{array}{cccc} 1 & 0 & 0 & 0\\ 0 & 1 & 0 & 0\\ 0 & 0 & 1 & 0\\ 0 & 0 & 0 & 1 \end{array}\right] \end{array}\;H_{r,d}=\;\left[\begin{array}{cccc} h_{1} & 0 & 0 & h_{2}\\ h_{2} & h_{1}j & 0 & 0\\ 0 & h_{2} & -h_{1} & 0\\ 0 & 0 & h_{2} & -h_{1}j \end{array}\right]$$

(34) $$H_{{eff}_{r,d}}^{rect}=\left(F_{N}⊗I_{M}\right)H_{r,d}\left(F_{N}^{H}⊗I_{M}\right)$$

(35) $$H_{{eff}_{r,d}}^{rect}=\;\frac{1}{\sqrt{2}}\left[\begin{array}{cccc} 1 & 0 & 1 & 0\\ 0 & 1 & 0 & 1\\ 1 & 0 & -1 & 0\\ 0 & 1 & 0 & -1 \end{array}\right]\;\;\;\times \left[\begin{array}{cccc} h^{1} & 0 & 0 & h_{2}\\ h_{2} & h^{1}j & 0 & 0\\ 0 & h_{2} & -h^{1} & 0\\ 0 & 0 & h_{2} & -h^{1}j \end{array}\right]\;\;\times \frac{1}{\sqrt{2}}\left[\begin{array}{cccc} 1 & 0 & 1 & 0\\ 0 & 1 & 0 & 1\\ 1 & 0 & {\;\;\;\;\;z}^{-2} & 0\\ 0 & 1 & 0 & z^{-2} \end{array}\right]$$

(36) $$H_{{eff}_{r,d}}^{rect}=\left[\begin{array}{cccc} 0 & h_{2} & h_{1} & 0\\ h_{2} & 0 & 0 & h_{1}j\\ h_{1} & 0 & 0 & -h_{2}\\ 0 & h_{1}j & h_{2} & 0 \end{array}\right]$$

In vectorized form, the received signal from the relay to the destination in the delay-Doppler domain can be written for the rectangular waveform:

(37) $${\bar{y}}_{r,d}=H_{{eff}_{r,d}}^{rect}\bar{x}+\bar{\tilde{n}}$$

## 3. End-to-End BER Calculation for the Proposed SDF Scheme

The system model expressions for the three channels are given below, respectively, for the delay-Doppler domain:

(38) $${\bar{y}}_{s,d}=H_{{eff}_{s,d}}^{rect}\bar{x}+{\bar{\tilde{n}}}_{s,d}$$

(39) $${\bar{y}}_{s,r}=H_{{eff}_{s,r}}^{rect}\bar{x\;}+{\bar{\tilde{n}}}_{s,r}$$

(40) $${\bar{y}}_{r,d}=H_{{eff}_{r,d}}^{rect}\bar{x\;}+{\bar{\tilde{n}}}_{r,d}$$

In our proposed method, we calculated the end-to-end bit error rate (BER) at the destination with OTFS waveform transmissions by implementing the SDF scheme, as presented below.

Using the total probability formula, the end-to-end BER at the destination can be expressed as follows:

(41) $$\mathrm{Pr}\left(e\right)=\mathrm{Pr}\left(e\cap \;\emptyset \right)+\mathrm{Pr}\left(e\;\cap \bar{\emptyset }\;\right)$$

where the event ∅ indicates the error at relay, the event $\bar{\phi }$ indicates no error at relay, and the event e indicates the end-to-end error at the destination. Then, the total probability formula considering conditional probabilities is

(42) $$\mathrm{Pr}\left(e\right)=Pr(e/\emptyset )\mathrm{Pr}\left(\emptyset \right)+\mathrm{Pr}(e/\bar{\emptyset })\mathrm{Pr}\left(\bar{\emptyset }\right)$$

where Pr(e/∅), related with Equation (38), is the probability of error at the destination in event error at relay; $\mathrm{Pr}\left(\emptyset \right)$, related with Equation (39), is the probability of error at relay; $\mathrm{Pr}(e/\bar{\emptyset })$ is the probability of error at the destination when the relay decodes accurately and $\mathrm{Pr}\left(\bar{\emptyset }\right)$ is the probability of no error at relay.

The probability of error at relay at high SNR is assumed to be zero as in below. Hence, the probability of no error at relay at high SNR is assumed to be one, as in below:

$$\mathrm{Pr}\left(\emptyset \right)\approx 0$$

$$\mathrm{Pr}\left(\bar{\emptyset }\right)\;\approx 1-Pr(\emptyset )\approx 1$$

Then, Equation(42) is transformed to the equation below:

(43) $$\mathrm{Pr}\left(e\right)\approx \mathrm{Pr}(e/\emptyset )Pr(\emptyset )+Pr(e/\bar{\emptyset })$$

Each model in Equations (38)–(40) can be accepted as the same as the classical multiple-input multiple-output (MIMO) system model structure with vector–matrix notation because the channel matrices are square matrices with *MN × MN* dimensions in all structures.

The approximate average BER expression for a Zero Forcing (ZF) MIMO system with BPSK modulation in a Rayleigh fading channel is presented as

(44) BERMIMO=2L−1L(12SNR)L

where SNR is the signal-to-noise ratio at the receiver, L=r−t+1 in which *r > t* is the diversity order of ZF, *r* is the number of receiver antennas and *t* is the transmit antennas in MIMO systems [26].

The approximate average BER *txt* MIMO (r = t and L = 1) channel in Rayleigh fading with ZF equalization is the same as the BER derived for the 1 × 1 (SISO) channel in Rayleigh fading, which is given as

(45) $${BER}_{SISO}\approx \frac{1}{2SNR}$$

where *L* equals to 1. Because the channel for the symbol transmitted from each spatial dimension is similar to a 1 × 1 (SISO) Rayleigh fading channel, Equation (44) is transformed to Equation (45) when L = 1 is taken into account.

Theoretical approximate average BER expressions for Pr(e/∅) and $\mathrm{Pr}\left(\emptyset \right)$ can be obtained from the source-to-destination transmission given in Equation (38) and the source-to-relay transmission given in Equation (43), respectively. Furthermore, the theoretical approximate average BER expression for the $\mathrm{Pr}(e/\bar{\emptyset })$ can be obtained using the concatenated single-input multiple-output (SIMO) model given below because the transmitted signal is the same but the received signal is different in each transmission.

(46) $$\left[\begin{array}{c} {\bar{y}}_{s,d}\\ {\bar{y}}_{r,d} \end{array}\right]=\left[\begin{array}{c} H_{{eff}_{s,d}}^{rect}\\ H_{{eff}_{r,d}}^{rect} \end{array}\right]\bar{x\;}+\left[\begin{array}{c} {\bar{\tilde{n}}}_{s,d}\\ {\bar{\tilde{n}}}_{r,d} \end{array}\right]$$

This model can be regarded as the same as the MIMO system model structure in which r > t. Therefore, the approximate average BER expression for this model can be obtained using Equation (47).

After obtaining $\mathrm{Pr}(e/\emptyset ),\;Pr(\emptyset )\;and\;Pr(e/\bar{\emptyset })$, the end-to-end BER calculation at the destination is obtained, as given below, using Equation (43):

(47) Pr⁡e=12SNRs,d×12SNRs,r+2L−1L(12SNRConcatanated)L

## 4. Performance Evaluation

In this section, the BER performance of the proposed SDF-OTFS scheme is evaluated and presented. Evaluations and simulations are performed in MATLAB R2018a.

P = M = N = 2 and BPSK modulation are also used in all simulations. Obtained *H* and $H_{eff}^{rect}$ matrices for the source-to-destination channel and the source-to-relay channel in Section 2 are used exactly the same. Maximum likelihood (ML) detection is used at the relay and the destination, since study [1] expressed that ML detection is convenient and easy to implement for the small numbers of MN in OTFS receivers.

**Figure 2:**
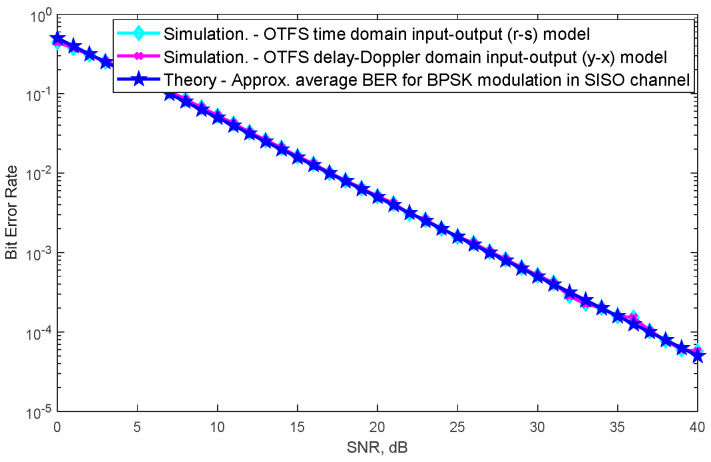
BER performances of OTFS time domain model, OTFS delay-Doppler domain model and theoretical approximate average BER in the SISO channel.

Figure 2 shows the BER performance of different SISO-OTFS input/output models according to the SNR values with the abovementioned parameters. Simulations have been completed with 10^6^ iterations. In each iteration, bit errors are taken into account by regenerating the new random transmitted bit sequence, channel coefficient gains and noises added at the receiver. The first of the different models is the OTFS time domain model in which there is a direct transmission between the transmitter and receiver antennas given in Equation (3). The *H* channel matrix is used, and the simulated SNR-BER graph is obtained according to this model. The second of the different models is the model known as the OTFS delay-Doppler domain model. The $H_{eff}^{rect}$ channel matrix given in Equation (8) is used, and the simulated SNR-BER graph is obtained according to this model. From Figure 2, it is understood that when these two channel models and the theoretical approximate average BER formula in Equation (45) are used, all of their SNR-BER performances overlap and are exactly the same. Furthermore, the same SNR-BER performances are also obtained for similar parameters of SISO-OTFS (Figure 4 of [27] and Figure 11 of [1] from the literature).

**Figure 3:**
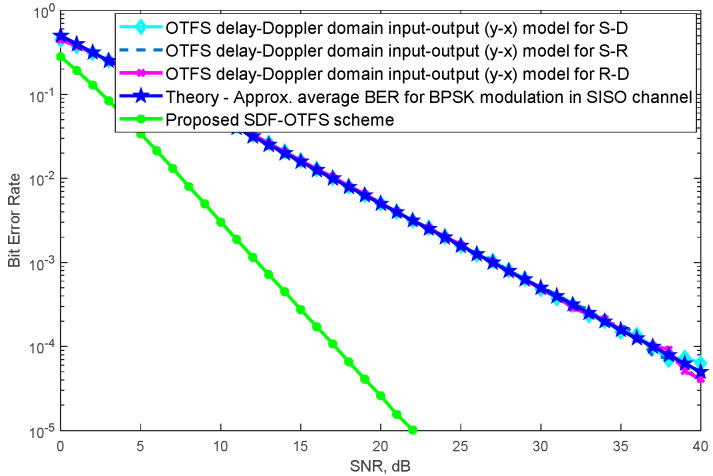
BER performance of the proposed SDF-OTFS scheme.

In Figure 3, the BER performance of the proposed SDF-OTFS scheme is presented. Simulations were completed according to the end-to-end probability calculations given in the previous section. As shown in Figure 3, the proposed SDF-OTFS scheme outperforms the point-to-point SISO-OTFS models considered in non-cooperative cases. For the non-cooperative case, we can consider source-to-destination (S-D) transmissions. This is the case where there is no relay, and only point-to-point transmission occurs. The performance result obtained is the same as in Figure 2. For verification purposes, different point-to-point SISO transmissions, such as source-to-relay (S-R), relay-to-destination (R-D) transmissions and transmissions with theoretical approximate average BER obtained from the SISO channel are also included and their performance results are given as in Figure 2. It was shown that the proposed SDF-OTFS model offers better quality of communication service against the non-cooperative case. For example, while 10^−5^ BER has been obtained at 22 dB SNR with the proposed SDF-OTFS scheme, nearly 4 × 10^−3^ BER has been obtained at 22 dB SNR with one of the SISO-OTFS models. To achieve 10^−4^ BER performance, a 37 dB SNR is required in one of the SISO-OTFS models, while a 17 dB SNR is sufficient for the proposed SDF-OTFS scheme. For the 10^−4^ BER performance, an approximately 100-times power efficiency is achieved when the proposed SDF-OTFS scheme is used instead of the point-to-point SISO-OTFS model.

## 5. Conclusions

In this study, to the best of authors’ knowledge, it has been addressed for the first time in the literature that high performance is achieved for the OTFS waveform transmission with an SDF cooperative communication scenario. In this context, by establishing a cooperative communication model consisting of a base station/source and a traffic sign/relay, end-to-end BER expressions for a smart vehicle/destination moving at a constant speed has been derived. BER analysis was performed with this SDF scheme and it was shown that a superior SNR-BER performance was achieved compared to the point-to-point SISO-OTFS model. With the proposed SDF-OTFS model, the door to better communication quality, low probability of outage and high energy efficiency has been opened for IoV applications that will be widely used in the future.

As future works, in order to reduce total power consumption which is a challenge for our method, an optimum power allocation method for base station/source and a traffic sign/relay can be developed to minimize the probability of end-to-end BER in the data (internet) communication of a smart vehicle moving at a constant speed. In this way, we can indirectly contribute to many areas such as reducing traffic congestion and accidents and saving fuel/energy by increasing the communication quality of smart vehicles, which would be an IoT or IoV element.

## Data Availability

Data sharing is not applicable.
